# Rowing on the Schuylkill, Damming on the Yangtze

**DOI:** 10.3201/eid1307.000000

**Published:** 2007-07

**Authors:** Polyxeni Potter

**Affiliations:** * Centers for Disease Control and Prevention, Atlanta, Georgia, USA

**Keywords:** Thomas Eakins, American naturalism, water-related disease, water sports, rowing, American art, art and emerging diseases, humanities and science, art commentary, about the cover

**Figure:**
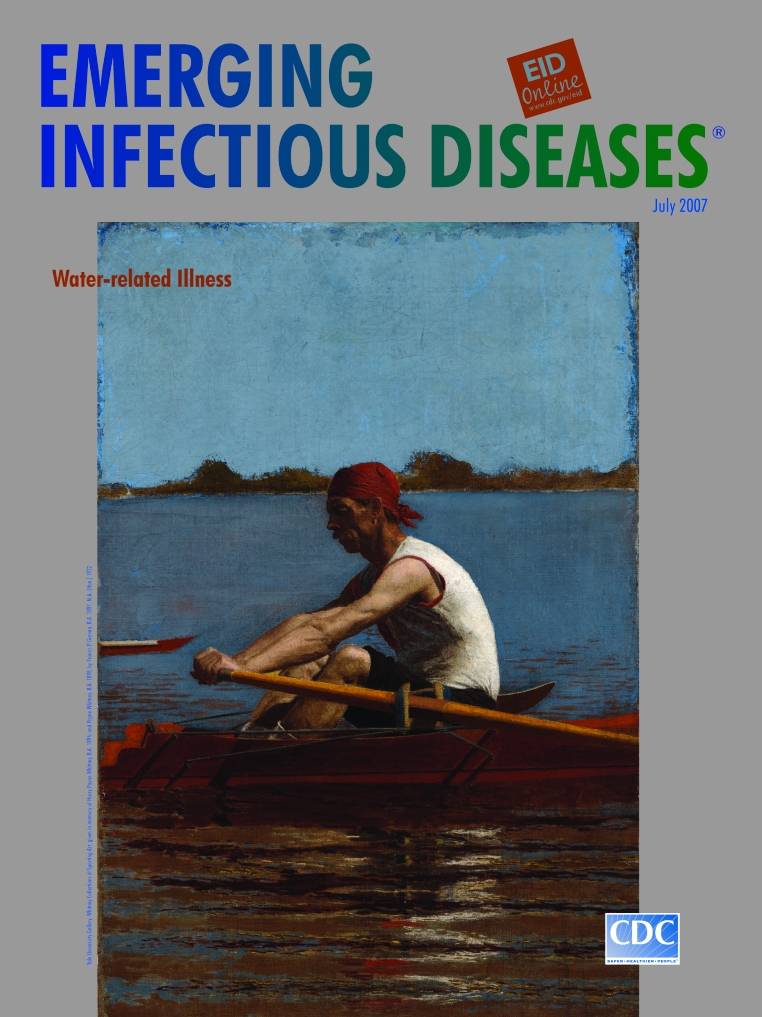
**Thomas Eakins (1844–1916). John Biglin in a Single Scull (1874).** Oil on canvas (61.9 cm × 40.6 cm). Yale University Art Gallery. Whitney Collections of Sporting Art, given in memory of Harry Payne Whitney, BA 1894 and Payne Whitney, BA 1898, by Francis P. Garvan, BA 1897, MA (Hon.) 1922

“Should the he-painters draw the horses and bulls, and the she-painters…the mares and cows?” asked Thomas Eakins, when critics derided his use of nude models in the presence of women art students (*1*). Eakins made no bones about his teaching practices and no compromises in his pursuit of artistic excellence. So he was viewed as radical and irascible, and although his art was widely discussed and exhibited, he did not see commercial success during his lifetime—he only sold 30 paintings.

A man ahead of his time, Eakins was born in Philadelphia, the son of a calligrapher, who nurtured his artistic talent and taught him the value of exacting detail. “I was born July 25, 1844. My father’s father was from the north of Ireland of the Scotch Irish. On my mother’s side my blood is English and Hollandish. I was a pupil of Gérôme (also of [portrait painter] Bonnat and Dumont, [the] sculptor). I have taught in life classes and lectured on anatomy continuously since 1873. I have painted many pictures and done a little sculpture….I believe my life is all in my work,” he wrote (*2*).

He studied drawing at the Pennsylvania Academy of Fine Arts and anatomy at Jefferson Medical College, traveled to Paris to attend the École des Beaux-Arts, and near the end of his studies, visited Spain “to see the pictures” (*2*). Despite his studies in Paris, he was most influenced by 17th-century Dutch and Spanish painters, particularly Diego Velázquez and Jusepe de Ribera. He lived the rest of his life in his beloved Philadelphia, following his own advice on achieving greatness: “remain in America to peer deeper into the heart of American life” (*3*).

Philadelphia and the Schuylkill River, which runs through it, held a special fascination for Eakins. He delighted in sailing, swimming, rowing, and all manner of outdoor activity before and after his travels abroad. Rowing, already a popular sport, attracted large crowds in the 1850s, when several rowing clubs formed the Schuylkill Navy, now the oldest amateur athletic governing body in the United States. As Eakins began his career and sought subjects from his immediate surroundings, he got caught up in the excitement of the sport, becoming one of the first artists to portray rowers in action. Sometimes he placed himself in the pictures and inscribed his name on the scull.

His painting of the human form, encouraged during his studies from nude models in Paris, was all but stifled by local culture, but the semi-nude athletic figure was socially acceptable. He produced nearly 30 rowing pictures from 1871 to 1874, at first painting his childhood friend Max Schmitt and later the Biglin brothers, a pair of celebrity rowers from New York. Still, reviews in the Philadelphia Inquirer were not glowing, “The artist, in dealing so boldly and broadly with the commonplace in nature, is working upon well-supported theories, and, despite somewhat scattered effect, gives promise of a conspicuous future” (*4*).

As teacher and later director of the Pennsylvania Academy of the Fine Arts, he introduced anatomy, dissection, and scientific perspective into the curriculum, revolutionizing art instruction. But he also scandalized school authorities with the use of nude models and was forced to resign in 1886. He continued to paint. “I will never have to give up painting, for even now I could paint heads good enough to make a living anywhere in America” (*5*). Later he was recognized for his formidable talent and was elected to the National Academy of Design. Provocative behavior though continued to damage his reputation, “My honors are misunderstanding, persecution, and neglect” (*5*).

Eakins’ approach to painting relied on close observation. He rejected embellishment and sentimentality and was the only artist, his friend Walt Whitman said, “who could resist the temptation to see what [he] think[s] ought to be rather than what is.” During the latter part of his career, he focused on portraiture: studies of relatives, friends, and persons accomplished in the sciences and other disciplines. The subject of a fine portrait in 1888, Whitman called Eakins “not so much a painter, as a force” (*6*). Unlike his contemporaries James McNeill Whistler, John Singer Sargent, and William Merritt Chase, popular society portraitists, Eakins painted his subjects with uncompromising realism and meticulous precision, which lent them a somber, aged, sometimes unflattering, aspect. While Chase’s studio was an atelier, Eakins joked, his own was a workshop.

One of his portraits, The Gross Clinic, widely acclaimed as the greatest American painting of the 19th century, depicts surgeon Samuel D. Gross performing surgery, instructing students, and training assistants to remove bone from the leg of an anesthetized patient. The portrait scandalized Victorian society. “It is a picture that even strong men find it difficult to look at long, if they can look at it at all”; wrote The New York Tribune, “and as for people with nerves and stomachs, the scene is so real that they might as well go to a dissecting room and have done with it” (*7*).

In John Biglin in a Single Scull, on this month’s cover, Eakins brought to bear his personal experience as rower and knowledge of the muscles involved. John Biglin, a “physical specimen…about as near perfect as can be found,” dominated the rowing scene in the 1860s and 70s (*8*). Sculpted as in relief, the figure is focused and intense, muscles terse, shoulders rounded. The composition is economical and accurate, from the sports hero’s facial features to the slightly worn wooden thole pin that held the oar in place for rowing. John Biglin, the quintessential outdoorsman equivalent of Samuel Gross the heroic physician!

Water activities continue on the Schuylkill and elsewhere, and the excitement of rowing remains undiminished as does the enjoyment of art. Our close relationship with water, far more complex than Eakins’ luminous river would suggest, has only become closer with better understanding of biology. In the 1850s, while rowing was becoming popular in Philadelphia, John Snow, the “father of epidemiology,” was investigating the water supply and sewage disposal in South London and finding that cholera is waterborne (*9*).

More than a hundred years later, diarrhea is the leading cause of childhood deaths in places that must rely on drinking water contaminated with pathogens (*10*). Invasive water organisms are spreading fast around the globe, damaging agriculture (*11*). And human activities, such as the Three Gorges Dam construction across the Yangtze River in People’s Republic of China, are threatening changes in ecology and setbacks in schistosomiasis control (*12*).

Water quality has environmental and social components. It is like good painting, which Eakins believed, extends beyond the geometry of landscape and the refraction of light on the waves to provide full understanding. Or, as he put it, “You can see what o’clock it is afternoon or morning if it’s hot or cold winter or summer and what kind of people are there and what they are doing and why they are doing it” (*13*).
